# Detection of Silent Type I Choroidal Neovascular Membrane in Chronic Central Serous Chorioretinopathy Using En Face Swept-Source Optical Coherence Tomography Angiography

**DOI:** 10.1155/2017/6913980

**Published:** 2017-12-04

**Authors:** Magdy Moussa, Mahmoud Leila, Hagar Khalid, Mohamed Lolah

**Affiliations:** ^1^Ophthalmology Department, Faculty of Medicine, Tanta University, Tanta, Egypt; ^2^MEDIC Eye Center, Tanta, Egypt; ^3^Retina Department, Research Institute of Ophthalmology, Giza, Egypt; ^4^Ophthalmology Department, Faculty of Medicine, Alexandria University, Alexandria, Egypt

## Abstract

**Purpose:**

To evaluate the efficacy of SS-OCTA in the detection of silent CNV secondary to chronic CSCR compared to that of FFA and SS-OCT.

**Patients and Methods:**

A retrospective observational case series reviewing the clinical data, FFA, SS-OCT, and SS-OCTA images of patients with chronic CSCR, and comparing the findings. SS-OCTA detects the CNV complex and delineates it from the surrounding pathological features of chronic CSCR by utilizing the blood flow detection algorithm, OCTARA, and the ultrahigh-definition B-scan images of the retinal microstructure generated by swept-source technology. The bivariate correlation procedure was used for the calculation of the correlation matrix of the variables tested.

**Results:**

The study included 60 eyes of 40 patients. Mean age was 47.6 years. Mean disease duration was 14.5 months. SS-OCTA detected type 1 CNV in 5 eyes (8.3%). In all 5 eyes, FFA and SS-OCT were inconclusive for CNV. The presence of foveal thinning, opaque material beneath irregular flat PED, and increased choroidal thickness in chronic CSCR constitutes a high-risk profile for progression to CNV development.

**Conclusion:**

Silent type 1 CNV is an established complication of chronic CSCR. SS-OCTA is indispensable in excluding CNV especially in high-risk patients and whenever FFA and SS-OCT are inconclusive.

## 1. Introduction

Theories of pathogenesis of central serous chorioretinopathy (CSCR) suggest that the retinal pigment epithelium- (RPE-) Bruch's membrane complex is compromised by shear stress caused by accumulating fluid that egresses from the choroidal circulation due to choroidal vascular hyperpermeability and consequent increased choroidal hydrostatic pressure [1–6]. Chronic CSCR is defined as persistent symptoms for at least 6 months from the onset of acute attack or persistent subretinal fluid associated with retinal pigment epitheliopathy and leaking points on fundus fluorescein angiography (FFA) [7]. Protracted contact between tenacious subretinal fluid and the RPE induces a spectrum of pathological changes in the RPE cell layer that includes atrophy, hypertrophy, and hyperplasia eventually leading to RPE cell dysfunction. The spectrum is known collectively as sick RPE syndrome and is considered the hallmark of chronic CSCR [7, 8]. The coexistence of choroidal vascular hyperpermeability, breached RPE-Bruch's complex, and chronic course purportedly incites the development of choroidal neovascular (CNV) membrane [9–12]. Detection of type 1 CNV secondary to chronic CSCR could be challenging using conventional angiography techniques, namely, FFA and indocyanine angiography (ICG), due to overlapping fluorescein leakage patterns and ICG fluorescence patterns of both pathologies [13–15]. On the other hand, optical coherence tomography (OCT) could yield inconclusive results even when deploying modern versions that provide ultrahigh-definition images acquired by swept-source (SS-OCT) technology. The reason is that pathological features associated with chronic CSCR include the thickened irregular RPE layer, in addition to subretinal deposits composed of shed photoreceptor outer segments and subretinal and sub-RPE lipoproteinaceous clumps derived from long-standing serous fluid. These features could have backscattering light intensity properties that are similar to those of the neovascular or fibrovascular components of the CNV, rendering both pathologies virtually indistinguishable from each other [13, 16]. The recently introduced swept-source OCT angiography (SS-OCTA) helped disentangle the perplexity of CNV underlying chronic CSCR by relaying information on the neovascular network that is unique for CNV. The SS-OCTA technology incorporates a blood flow detection algorithm, OCTARA, that is capable of visualizing the superficial retinal capillaries and foveal avascular zone, inner and outer retinal vascular plexuses, choriocapillaris, and larger choroidal vasculature in vivo without the need for contrast injection [17]. In addition, SS-OCTA utilizes the features of merged SS-OCT technology including long-wavelength (1050 nm) scanning light, less susceptibility-to-sensitivity roll-off, and ultrahigh-speed image acquisition. These features enabled deeper penetration and superior axial resolution and generation of ultrahigh-definition B-scan images of the retinal microstructure [18–22]. Thus, SS-OCTA could detect the CNV complex and delineate it from the surrounding pathological features of chronic CSCR particularly where FFA and SS-OCT alone reveal inconclusive results. This notion has been corroborated by several studies that reported the use of OCTA technology to detect CNV secondary to chronic CSCR, which was unrevealed by conventional imaging modalities [9, 11, 13, 23]. The current study aims to evaluate the efficacy of SS-OCTA in the detection of silent CNV secondary to chronic CSCR and to further compare the results with those of conventional FFA and with those of SS-OCT.

## 2. Patients and Methods

### 2.1. Patients

This is a retrospective observational case series in which we reviewed the clinical data, FFA, SS-OCT, and SS-OCTA images of all consecutive patients diagnosed with chronic CSCR in a private practice from September 2015 to June 2017, and compared the findings.

### 2.2. Inclusion Criteria

All patients included in the study had typical features of chronic CSCR on biomicroscopic examination (diffuse retinal pigment epitheliopathy, neurosensory and/or RPE detachment, and subretinal deposits), on FFA (early leaking point(s) at the level of the RPE, pooling of dye in single or multiple areas of PED, and alternating areas of hyper- and hypofluorescence caused by RPE alteration), and on SS-OCT (neurosensory detachment, solitary or multiple PED(s), irregularly thickened RPE layer, hyperreflective amorphous subretinal and/or sub-RPE deposits, and increased choroidal thickness). Patients were required to have a disease duration of more than 6 months to be eligible for the study. All recruited patients were treatment naïve at the time of enrollment.

### 2.3. Exclusion Criteria

The exclusion criteria included concomitant ocular diseases that could cause localized serous detachment of the macula, such as diabetic retinopathy, vascular occlusion, age-related macular degeneration, and optic pit maculopathy, or hereditary ocular diseases and patients presenting initially with documented concomitant choroidal neovascularization or media opacity that was dense enough to preclude sufficient image quality for reliable interpretation. Any patient who received treatments for chronic CSCR that might have altered the features of the disease or masked preexisting CNV including laser treatment, anti-VEGF agents, photodynamic therapy (PDT), or mineralocorticoids were excluded from the study.

## 3. Methods

### 3.1. FFA

FFA images were obtained using a Topcon TRC 50DX fundus camera (Topcon Corporation, Tokyo, Japan).

### 3.2. SS-OCT

SS-OCT images were acquired using the DRI OCT Triton machine version 10.11 (Topcon Corporation, Tokyo, Japan). The machine incorporates the swept-source technology, which utilizes an infrared (1050 nm) laser source and analog-to-digital acquisition mode that minimizes variation in sensitivity with depth (sensitivity roll-off) allowing deeper penetration and superior axial resolution. The infrared laser operates at a scanning speed of 100,000 A-scans/second. This ultrahigh acquisition speed enables dense raster scanning to acquire high volumetric data generating ultrahigh-definition B-scan images. The routine protocol used for scanning each patient consisted of a radial scan consisting of 12 radial lines (1024 A-scans × 12) (each line is 9 mm or 12 mm in length to include the entire lesion) centered onto the fovea, horizontal and vertical line scans (1024 A-scans, 9.00 mm) centered onto the fovea, and a 3D scan (512 A-scans × 256 scan lines). The central foveal thickness (CFT) value was obtained from a macular thickness map displayed as ETDRS grids. The choroidal thickness in the subfoveal area was measured using calipers from the outer boundary of the RPE to the inner boundary of the sclera.

### 3.3. SS-OCT Angiography (SS-OCTA) and OCTARA Algorithm

OCTARA (optical coherence tomography angiography ratio analysis) (Topcon Corporation, Tokyo, Japan) is a blood flow detection algorithm that uses decorrelation motion contrast between rapidly repeated SS-OCT B-scans to visualize blood flow in vivo without the need for contrast injection. This OCTA implement benefits from being merged with SS-OCT technology as the deeper penetration of the infrared wavelength allows segmentation of different layers of the ocular fundus. In case CNV is present, it is possible to generate depth-resolved images of the neovascular lesion and its location in relation to the RPE-Bruch's-choriocapillaris complex. Acquired scans are displayed simultaneously as separate en face images of 3 retinal layers (superficial capillary plexus, deep capillary plexus, and outer retina) and the choriocapillaris. It is worthy of note that the OCTARA algorithm generates OCTA images by registering B-scan repetition at each scan location and then computing a ratio-based result between corresponding image pixels. This method preserves the integrity of the OCT spectrum and does not result in compromised axial resolution, an inherent disadvantage of other OCTA technologies.

### 3.4. Image Acquisition

OCTA acquisition protocol in the macular region consisted of a 3 × 3 mm^2^ area centered onto the fovea for maximum resolution of the lesion examined. Whenever the lesion extended beyond the image border, a 4.5 × 4.5 mm^2^, 6 × 6 mm^2^, or 9 × 9 mm^2^ area was used to include the entire extent of the lesion. By default, the integrated software (IMAGEnet 6 ophthalmic data system) deploys automated segmentation to delineate the lesion and its location in relation to the RPE-Bruch's membrane-choriocapillaris complex. In cases of significant disorganization of retinal layers as in large PED or sizeable subretinal fluid or deposits, the integrated automated segmentation feature failed to detect the correct boundaries of the lesion and we had to resort to manual adjustment of the segmentation slab. To perform manual segmentation, the operator manually places two segmentation lines at sequential depths guided by corresponding SS-OCT images to reveal the maximum extent of the lesion and its actual location.

### 3.5. Color Coding

The SS-OCTA software generates a color-coded flow density map of the retinal superficial and deep capillary plexuses, the outer retina, and the choriocapillaris that could be displayed individually or as a composite montage of all 4 layers. Bright red color represents areas of dense vascular flow, whereas dark blue represents areas devoid of blood flow. Intermediate color shades represent variable grades of flow. This density map helps to highlight abnormal vascular flow within a neovascular complex relative to the surrounding avascular outer retina or the choriocapillaris.

### 3.6. SS-OCTA Interpretation

The SS-OCTA criteria for active CNV were defined as interlacing tiny capillaries, extensive arborization, vascular anastomosis, and looping, whereas the criteria for inactive CNV were defined as large linear vessels widely separated by dark spaces with no or minimal anastomosis and the presence of single or multiple feeder vessels supplying the neovascular complex.

All three imaging modalities were performed on the same day. Patient selection for enrollment and image interpretation were undertaken by an experienced retina specialist (MM). The study was performed in accordance with the tenets of the Declaration of Helsinki of 1975 (the 2013 revision). All patients received thorough explanation of the procedures entailed in the study and signed an informed consent before undertaking any of the imaging modalities described above. The consent included a statement that authorized the authors to publish patients' photos and data for research purposes in an anonymous manner that does not allow identification of the patient.

### 3.7. Statistical Analysis

For the calculation of the correlation matrix, we used the bivariate correlation procedure that computes Pearson's correlation coefficient. Correlation measures how variables are related. Two variables can be perfectly related, but if the relationship is not linear, Pearson's correlation coefficient is not an appropriate statistic. The results of the *r* value were checked on the *r* table to find out the significant level. Correlation coefficient (*R*) = (*N* ∑*XY* − ∑*X* ∑*Y*)/[SQR (*N*∑*X*^2^ − (∑*X*)^2^) (*N*∑*Y*^2^ − (∑*Y*)^2^)], where *X* is the independent variable and *Y* is the dependent variable. The coefficient of determination (*R*^2^) procedure computes relative contribution of independent variable (*X*) to the dependent variable (*Y*).

## 4. Results

### 4.1. Patients' Characteristics

The study included 60 eyes of 40 patients (34 men and 6 women) with a mean age of 47.6 years (range 26–66; SD 10.5). Chronic CSCR was bilateral in 20 patients (50%). Mean best-corrected visual acuity (BCVA) was 0.4 logMAR (range 0–1.5 logMAR; SD 0.4). Mean disease duration was 14.5 months (range 6–28 months; SD 6) (Table 1).

### 4.2. Chronic CSCR Features

Residual subretinal fluid was present in 44 eyes (73.3%). In the remaining 16 eyes (27%), subretinal fluid was more extensive in the form of neurosensory detachment. Mean CFT by SS-OCT was 242 *μ* (range 102-725 *μ*; SD 110). Subretinal deposits were encountered in 39 eyes (65%). PED was encountered in 55 eyes (92%), of which 46 eyes (84%) had the thick flat irregular variant, whereas 9 eyes (16.3%) had the dome-shaped smooth regular variant. Sub-RPE deposits were detected in 35 (58%) eyes. Disrupted outer retinal layers including external limiting membrane (ELM) and inner segment/outer segment (IS/OS) junction were present in 49 eyes (82%). Foveal thinning was detected in 14 eyes (23%). One patient (1.6%) had choroidal excavation. Choroidal thickening with dilated choroidal vessels were detected in 50 (83%) out of 60 eyes included in the study. Mean subfoveal choroidal thickening was 412 *μ* (range 196–737 *μ*; SD 113). Mean subfoveal choroidal thickening of the contralateral eye of the 20 patients who had unilateral chronic CSCR was 354 *μ* (range 235–526 *μ*; SD 86) (Table 2).

### 4.3. Silent Type 1 Choroidal Neovascularization

SS-OCTA imaging detected type 1 CNV formation in 5 (8.3%) out of 60 eyes; one of these eyes had polypoidal choroidal vasculopathy (PCV) variant of type 1 CNV. In all 5 cases, FFA and SS-OCT findings were inconclusive for CNV.

### 4.4. Correlation between Chronic CSCR Features and CNV Development

For statistical analysis, the study population was classified into 2 groups. Group I included 55 eyes that were not complicated with CNV. Group II included 5 eyes that developed CNV. Subsequently, we proceeded to the assessment of the contribution of chronic CSCR features to the CNV-free status in group I and to the development of CNV in group II. The assessed features were PED (regular smooth or irregular flat), absence or presence of opaque material beneath flat PED, foveal thinning, disrupted outer retinal layers, choroidal excavation, subretinal deposits, sub-RPE deposits not related to PED, and increased choroidal thickness. Statistical analysis revealed that for group II, the total contribution of all studied features to CNV development was 65.6% in comparison to 11.1% of the contribution of the same factors to the CNV-free status in group I. Moreover, in group II, 3 major features were identified as statistically significant predictors for CNV development, namely, foveal thinning (75.0), presence of opaque material beneath flat PED (11.22), and increased choroidal thickness (7.84) (Table 3).

## 5. Case Reports

### 5.1. Case 1

**Figure 1:**
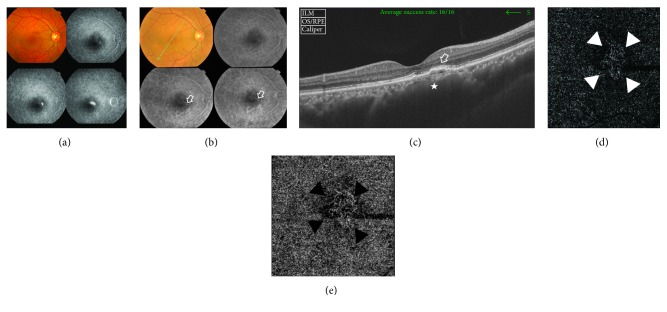
(a) The colored photo and FFA of the right eye of a 52-year-old male during acute CSCR. The macular area shows neurosensory detachment, measuring approximately 3 DD. Corresponding FFA shows a typical smoke-stack leaking pattern. (b) The colored photo and FFA of the same eye 2 years later. The posterior pole shows minimal retinal pigment disturbance in the inferior portion of the macular area along with subretinal deposits. On FFA, the old site of acute leakage demonstrates early pinpoint hyperfluorescence with increasing intensity through late frames suggestive of chronic point of leakage (open arrow). The area of pigment epitheliopathy seen in the colored photo shows hyperfluorescence due to window defect. (c) The corresponding SS-OCT image in a radial scan mode shows shallow PED with surface undulations and hyperreflective sub-RPE deposits (open arrow). There is associated disruption of ELM and IS/OS layers in the vicinity of the PED. Note the generalized dilatation of the choroidal vessels that is most marked in the area beneath the PED (star). Subfoveal choroidal thickness is 737 *μ*. (d) The en face SS-OCTA image of the same eye taken at the level of the outer retina in a 6 mm × 6 mm field. Note the hyperintense signal caused by increased blood flow within the vascular network of tiny interlacing capillaries (white arrow heads). The neovascular complex is surrounded by a hypointense hollow zone intervening between the lesion and the surrounding normal outer retina that generates a hypointense signal due to the absence of blood flow and is displayed as a dark-grey background. (e) The en face SS-OCTA image of the same lesion at the level of the choriocapillaris (black arrow heads).

A 52-year-old male has bilateral chronic CSCR of approximately 2-year duration. His BCVA was 1 logMAR and 0.2 logMAR in the right and left eyes, respectively. FFA of the right eye when he first presented during the acute attack showed typical smoke-stack appearance. As the chronic stage of the disease ensued, the fundus showed RPE pigmentary disturbance in the macular area along with subretinal deposits. On FFA, the old site of acute leakage demonstrated early pinpoint hyperfluorescence with increasing intensity through late frames suggestive of chronic point of leakage. The area of pigment epitheliopathy seen in the colored photo showed hyperfluorescence due to window defect. The corresponding SS-OCT scan of the macular area showed flat irregular PED and sub-RPE heterogeneous deposits. Subfoveal choroid was markedly thickened (737 *μ*). FFA and SS-OCT were inconclusive for the presence of CNV. SS-OCTA of the same eye clearly demonstrated the decorrelation signal characteristic of blood flow within an abnormal vascular network of active CNV (Figure 1). SS-OCTA of the left eye was normal.

### 5.2. Case 2

**Figure 2:**
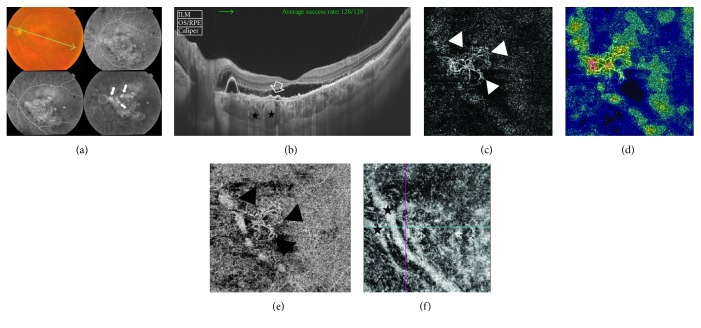
(a) The colored photo and FFA of the left eye of a 46-year-old male with bilateral chronic CSCR of approximately 1.5-year duration. The posterior pole shows diffuse retinal pigment epitheliopathy in the macular area measuring approximately 5 disc diameters (DD) in size along with shallow subretinal fluid and peripapillary PED. FFA shows a large hyperfluorescent area caused by window defect that corresponds to the area of retinal pigment epitheliopathy seen in the colored photo. In addition, the macular area shows multiple hyperfluorescent leaking points that gradually increase in intensity throughout later phases with a typical inkblot pattern of leakage (arrows). (b) SS-OCT image. A 12.0 mm radial scan is selected to include the peripapillary area. The scan shows subfoveal neurosensory detachment extending to the peripapillary area, which is associated with subretinal deposits. A single large regular smooth PED is seen in the peripapillary area. A subfoveal shallow PED with an undulating thickened surface and sub-RPE hyperreflective amorphous deposits is shown (open arrow). The subfoveal choroidal vessels are engorged (black stars). Subfoveal choroid thickness is 455 *μ*. (c) The en face SS-OCTA image of the same eye taken at the level of the outer retina in a 4.5 mm × 4.5 mm field. Note the hyperintense signal caused by increased blood flow within the CNV complex. The complex is composed of a network of tiny arborizing capillaries with frequent anastomosis and looping in the periphery of the lesion (white arrow heads). (d) The corresponding color-coded density map reflecting blood flow at the level of the outer retina. Note the entire background has variable shades of blue typical of avascular outer retina. The CNV complex stands out with its bright red signal indicating dense blood flow. (e) The en face SS-OCTA image of the same lesion at the level of the choriocapillaris (black arrow heads). (f) The en face SS-OCTA image after manual adjustment of the segmentation slab to a deeper position into the choroid to demonstrate two abnormally dilated choroidal vessels corresponding to increased subfoveal choroidal thickness on SS-OCT (black stars).

A 46-year-old male has bilateral chronic CSCR of approximately 1.5-year duration. His BCVA was 0.3 logMAR and 0.7 logMAR in the right and left eyes, respectively. Fundus examination of the left eye showed diffuse retinal pigment epitheliopathy in the macular area measuring approximately 5 disc diameters (DD) along with shallow neurosensory detachment. FFA of the same eye showed a large hyperfluorescent area caused by window defect that corresponds to the area of retinal pigment epitheliopathy seen in the colored photo. In addition, the macular area showed multiple hyperfluorescent leaking points that gradually increased in intensity throughout later phases with a typical inkblot pattern of leakage. SS-OCT of the same eye demonstrated neurosensory detachment along with subfoveal flat irregular PED associated with sub-RPE deposits and a single large regular smooth PED in the peripapillary area. Subfoveal choroid was thickened (455 *μ*) with markedly dilated large choroidal vessels. FFA and SS-OCT were inconclusive for CNV formation. SS-OCTA of the left eye revealed the characteristic hyperintense signal of blood flow within the interlacing capillary network with looping and vascular anastomosis that was indicative of active CNV (Figure 2). SS-OCTA of the right eye was normal.

### 5.3. Case 3

**Figure 3:**
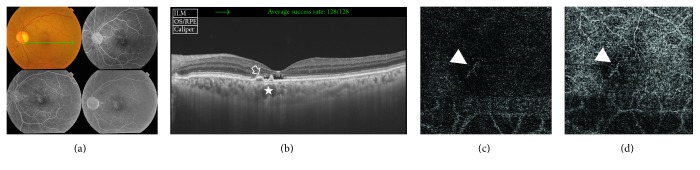
(a) The colored photo and FFA of the left eye of a 40-year-old male with left chronic CSCR of approximately 1-year duration. The posterior pole shows retinal pigment epitheliopathy in the macular area. FFA of the same eye shows multiple hyperfluorescent leaking points in the macular area that gradually increase in intensity throughout later phases resulting in a diffuse ill-defined hyperfluorescence pattern. (b) The corresponding SS-OCT image in a radial scan mode shows subfoveal shallow neurosensory detachment with subretinal deposits and associated disruption of ELM and IS/OS layers. Note the double-humped irregular PED in the parafoveal area with underlying opaque hyperreflective material (open arrow). The choroid vessels underneath the PED are engorged (star). Subfoveal choroidal thickness is 315 *μ*. (c) The *en face* SS-OCTA image of the same eye taken at the level of the outer retina in a 6 mm × 6 mm field. Note the hyperintense signal caused by increased blood flow within the CNV complex composed of tiny intertwining vascular loops (arrow head). (d) The en face SS-OCTA image of the same lesion at the level of the choriocapillaris.

A 40-year-old male has left chronic CSCR of approximately 1-year duration. His BCVA was 1 logMAR. The colored fundus photo of the left eye showed retinal pigment epitheliopathy in the macular area. FFA of the same eye showed hyperfluorescence due to a combination of window defect and multiple leaking points. SS-OCT of the same eye showed disruption of outer retinal layers with irregular flat PED formation and sub-RPE hyperreflective heterogeneous deposits and slightly thickened subfoveal choroid (315 *μ*). FFA and SS-OCT were inconclusive for CNV formation. SS-OCTA of the same eye showed the characteristic hyperintense signal of blood flow within the abnormal tiny capillary network that was indicative of active CNV formation (Figure 3).

### 5.4. Case 4

**Figure 4:**
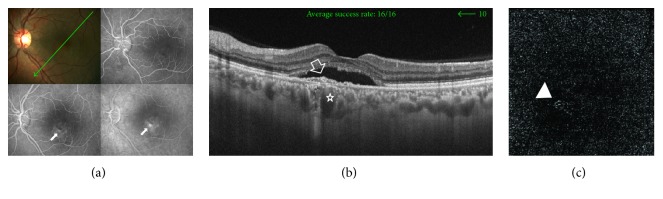
(a) The colored photo and FFA of the left eye of a 35-year-old male with chronic CSCR of approximately 2-year duration. The macular area shows shallow neurosensory detachment. Corresponding FFA shows hyperfluorescent leaking points (arrows). (b) The radial scan SS-OCT image of the same eye demonstrates subfoveal neurosensory detachment with subretinal hyperreflective deposits. A subfoveal flat irregular PED associated with sub-RPE hyperreflective material is seen (open arrow). Subfoveal choroidal thickness is 400 *μ*. Note the engorged choroidal vessels beneath the PED (star). (c) The en face SS-OCTA image of the same eye at the level of the outer retina in a 4.5 mm × 4.5 mm field. Note the characteristic hyperintense signal of vascular flow within tiny interlacing capillaries in the foveal area (arrow head).

A 35-year-old male has left chronic CSCR of approximately 2-year duration. His BCVA was 0.5 logMAR. The colored photo of the left eye showed shallow neurosensory detachment in the macular area. SS-OCT of the same eye demonstrated subfoveal neurosensory detachment with subretinal deposits. In addition, there was flat irregular PED associated with sub-RPE hyperreflective amorphous material. Subfoveal choroid was thickened (400 *μ*). SS-OCTA of the same eye revealed the characteristic hyperintense signal of blood flow within the tiny interlacing capillary network that was indicative of active CNV (Figure 4).

### 5.5. Case 5

**Figure 5:**
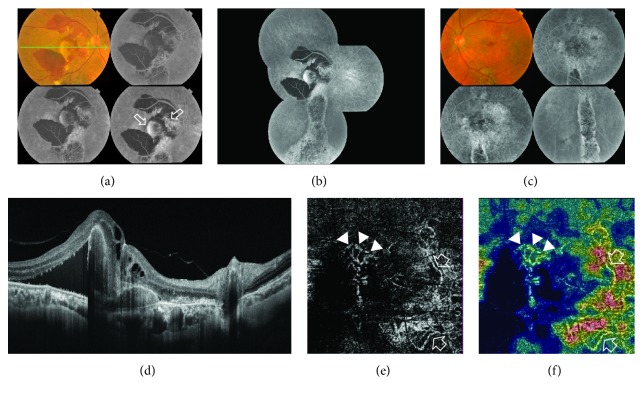
(a) The colored photo and FFA of the right eye of a 61-year-old male with bilateral chronic CSCR of approximately 2.5-year duration. The posterior pole shows a subretinal yellowish-white elevated lesion in the macular area measuring approximately 2 DD. The lesion is surrounded by extensive subretinal hemorrhage that extends beyond the temporal vascular arcades. On FFA, there were two distinct patterns of hyperfluorescence. The first pattern corresponded to the yellowish-white lesion seen in the colored photo and consisted of early hyperfluorescence that gradually increased in intensity due to pooling into PED. The second pattern consisted of early stippled hyperfluorescence that increased in intensity throughout the angiogram resulting in an ill-defined hyperfluorescent area (open arrows). Note the blocked fluorescence corresponding to subretinal hemorrhage in the colored photo. (b) The composite FFA of the right eye shows extensive retinal pigment epitheliopathy in the macular area, extending inferiorly due to gravitation of subretinal fluid giving rise to the characteristic teardrop sign (atrophic RPE track). (c) The colored photo and FFA of the left eye showing diffuse retinal pigment epitheliopathy and the characteristic teardrop sign. (d) The radial scan SS-OCT of the right eye demonstrates neurosensory detachment along with subretinal hyperreflective heterogeneous material, sizeable subfoveal PED, and sub-RPE hyperreflective material. (e) The SS-OCTA of the right eye at the level of the outer retina in a 4.5 mm × 4.5 mm field revealed the characteristic hyperintense signal of a subfoveal neovascular complex composed of a main trunk with branching smaller vessels each terminating in polyp-like configuration reminiscent of PCV (arrow heads). In addition, there was another juxtapapillary larger neovascular complex composed of dense capillary arborization. At least two feeder vessels could be identified (open arrows). (f) The corresponding color-coded density map reflecting blood flow at the level of the outer retina. Note the intense red signal denoting high flow within the neovascular complexes seen, contrasting with the blue background typical of avascular outer retina.

A 61-year-old male has bilateral chronic CSCR of approximately 2.5-year duration. His BCVA was 1.5 logMAR and 0.7 logMAR in the right and left eyes, respectively. The colored photo of the right eye showed a subretinal yellowish-white elevated lesion in the macular area, measuring approximately 2 DD and surrounded by subretinal hemorrhage. The posterior pole showed extensive retinal pigment epitheliopathy. On FFA, there were two distinct patterns of hyperfluorescence. The first pattern corresponded to the yellowish-white lesion seen in the colored photo and consisted of early hyperfluorescence that gradually increased in intensity due to pooling into PED. The second pattern consisted of early stippled hyperfluorescence that increased in intensity throughout the angiogram resulting in an ill-defined hyperfluorescent area. Other associated angiographic features were blocked fluorescence corresponding to subretinal hemorrhage and hyperfluorescence due to window defect corresponding to diffuse retinal pigment epitheliopathy. SS-OCT of the same eye demonstrated neurosensory detachment along with subretinal hyperreflective heterogeneous material, sizeable subfoveal PED, and sub-RPE hyperreflective material. The blocking effect of significant hemorrhage hindered exclusion of CNV formation using FFA and SS-OCT alone. SS-OCTA of the right eye revealed the characteristic hyperintense signal of a subfoveal neovascular complex composed of a main trunk with branching smaller vessels each terminating in polyp-like configuration reminiscent of PCV. In addition, there was another juxtapapillary larger neovascular complex composed of dense capillary arborization and feeder vessels (Figure 5). SS-OCTA of the left eye was normal.

## 6. Discussion

Choroidal neovascular membrane masquerading as chronic CSCR is an uncommon yet devastating complication that eventually leads to irreversible visual loss [10, 11, 24]. Therefore, early detection of CNV arising on top of chronic CSCR is pivotal in establishing the diagnosis and prompt initiation of therapy for vision salvage in those patients. Particularly, type 1 variant of CNV can present a diagnostic predicament using conventional imaging modalities [13–16].

In the current series, we combined SS-OCTA with SS-OCT and FFA for the detection of CNV in chronic CSCR cases. Our results revealed the presence of type 1 CNV in 8.3% of the study population, of which one eye developed PCV. This finding is comparable with that of Hage et al. [14] who reported a 5.8% incidence of type 1 CNV in a retrospective case series of 172 eyes. Similarly, a prospective observational cross-sectional study including 27 eyes with chronic CSCR by Filho et al. [23] detected CNV secondary to chronic CSCR in 8 out of 27 eyes (30%). Four eyes had type 1 CNV, whereas four eyes developed type 1 and type 2 mixed variant. El-Maftouhi et al. [13] reported a 58% incidence of CNV secondary to chronic CSCR. De Carlo et al. [11] detected CNV in 28.5% of 49 eyes with chronic CSCR. Our finding is further corroborated by two other series by Yannuzzi et al. [12] and Fung et al. [10] and a case report by Yang et al. [15] who detected CNV secondary to chronic CSCR. These authors used multimodal imaging exclusive of OCTA and reported type 1 CNV and its variant PCV either masquerading as chronic CSCR at presentation or developing over the course of follow-up.

The current study focused on the analysis of several characteristic features of chronic CSCR (Table 2) and their correlation with the development of CNV. Our results revealed 3 statistically significant major predictors in SS-OCT images for progression to neovascularization, namely, foveal thinning, presence of opaque material beneath irregular flat PED, and increased choroidal thickness. Accordingly, we could propose a high-risk profile of chronic CSCR for progression to type 1 CNV formation. In accordance with our findings, Hage et al. [14] detected the presence of flat irregular PED in all eyes with chronic CSCR that developed CNV. The authors coined the terms “optically filled” and “not optically filled” to describe OCT findings of the amorphous contents of the sub-RPE space beneath the PED. All eyes with CNV in their series had an optically filled sub-RPE space. Similarly, De Carlo et al. [11] classified patients into two groups: those with regular PED and those with irregular PED. The authors stated that the presence of irregular flat PED in chronic CSCR is a statistically significant risk factor for the development of CNV. The authors however did not correlate the content of the sub-RPE space with CNV in their results. Instead, they studied the relation between PED and sub- or intraretinal fluid and concluded that the presence or absence of fluid was not helpful in predicting the development of CNV in chronic CSCR. Our findings regarding increased choroidal thickness in patients with chronic CSCR are consistent with those of the published literature that corroborate the choroidal vascular hyperpermeability theory as the main etiological factor of the disease [5, 6, 25–27]. Moreover, in our series, increased choroidal thickness was a statistically significant contributing factor in the development of CNV in the 5 cases reported herein. In one of our cases (Figure 2), we were able to demonstrate a marked increase in the caliber of choroidal vessels using SS-OCTA projection of large choroidal vessels that corresponded to subfoveal increased choroidal thickness in SS-OCT. In accordance with our findings, El-Maftouhi et al. [13] found that all eyes, which developed CNV in chronic CSCR, had associated choroidal vascular hyperpermeability on ICG angiography, whereas all eyes that did not develop CNV had normal choroidal circulation. Similarly, Yang et al. [16] in a prospective series including 68 eyes with CSCR reported mean subfoveal choroidal thickness of 478 *μ*. It is worthy of note that in the current study, we detected increased choroidal thickness in the normal fellow eye of those patients who had unilateral chronic CSCR, though to a lesser extent than that in the affected eye (412 *μ* versus 354 *μ*). This finding is congruous with that of the study of Kim et al. [28] who reported increased choroidal thickness of unaffected eyes in patients with unilateral CSCR.

The important limitation of the current study is the lack of comparison between SS-OCTA and ICG angiography in assessing the choroidal circulation particularly that increased choroidal thickness is one of the 3 major predictors of progression to CNV formation according to our results. However, evidence from the literature supports the notion that increased choroidal thickness by SS-OCT is equivalent to choroidal vascular hyperpermeability [25] and that SS-OCTA projection of choroidal circulation corresponds to ICG in the evaluation of choroidal blood flow in patients with CSCR [29]. Another limitation is that the current study did not include a concurrent comparison group that included patients with chronic CSCR subjected to treatment with PDT and/or anti-VEGF agents. Comparing both groups would reveal important insights into the progression of chronic CSCR to CNV formation and the effect of treatment on the alteration of the high-risk chronic CSCR profile we proposed in the current study.

## 7. Conclusion

Silent type 1 CNV is an established complication of chronic CSCR. The presence of foveal thinning, irregular flat RPE associated with sub-RPE deposits, and increased choroidal thickness represents a high-risk profile that warrants exclusion of CNV formation using multimodal imaging protocol in which SS-OCTA is an integral component alongside conventional FFA and SS-OCT, particularly when both reveal inconclusive results. A combination of SS-OCT and SS-OCTA is a risk-free analogue to FFA in clinical situations where the latter is contraindicated.

## Figures and Tables

**Table 1:** Baseline patients' characteristics.

Baseline characteristics	*N* (%)
Male	34 (85)
Female	6 (15)
Age (years)	
<40	9 (22.5)
40–50	16 (40)
51–60	7 (17.5)
>60	8 (20)
Baseline BCVA (logMar)	
0–0.1	18 (30)
>0.1–0.3	14 (23.3)
>0.3–1	26 (43.3)
>1	2 (3.3)
Laterality	
Unilateral	20 (50)
Bilateral	20 (50)
Disease duration (months)	
6–12	25 (41.6)
>12–18	26 (43.3)
>18	9 (15)

**Table 2:** Chronic CSCR features in the study population.

Chronic CSCR features	*N* (%)
Residual subretinal fluid	44 (73.3)
Neurosensory detachment	16 (27)
PED (total number of eyes)	55 (92)
(i) Flat irregular PED	46 (84)
(ii) Dome-shaped smooth PED	9 (16.3)
Disrupted ELM-IS/OS layers	49 (82)
Foveal thinning	14 (23.3)
Choroidal excavation	1 (1.6)
Mean choroidal thickening (*μ*)	
(i) Affected eye(s)	412
(ii) Contralateral normal eye(s)	354

CSCR: central serous chorioretinopathy; ELM: external limiting membrane; IS/OS: inner segment/outer segment junction; PED: pigment epithelial detachment; *μ*: micron.

**Table 3:** Relative contribution (%) for studied chronic CSCR features in CNV development.

Feature	Group I (55 patients) = not complicated *N* (%)	Group II (5 patients) = complicated *N* (%)
RPED	9 (0.32)	0 (0.00)
IRPED	41 (2.02)	5 (0.66)
IRPED-T	12 (0.16)	0 (1.80)
IRPED-O	29 (1.02)	5 (11.22^∗∗^)
FATR	11 (13.18^∗∗^)	3 (75.00^∗∗^)
DOLR	44 (3.03)	5 (0.45)
CEXC	1 (0.26)	0 (0.45)
SRD	35 (2.56)	4 (0.00)
SRPED-EF	30 (0.07)	5 (0.45)
CT	45 (1.23)	5 (7.84^∗∗^)
Total relative contribution (%)	11.10	65.6

^∗∗^Significant at 1%. CEXC: choroidal excavation; CT: choroidal thickness; DOLR: disrupted outer retinal layers; FT: foveal thinning; IRPED: irregular flat PED; IRPED-T: irregular flat PED with translucent (empty) sub-RPE space; IRPED-O: irregular flat PED with opaque (filled) sub-RPE space; RPED: regular smooth PED; SRD: subretinal deposits; SRPED-EF: sub-RPE deposits not related to irregular flat PED.

## References

[1] Prünte C., Flammer J. (1996). Choroidal capillary and venous congestion in central serous chorioretinopathy. *American Journal of Ophthalmology*.

[2] Spaide R. F., Ryan E. H. (2015). Loculation of fluid in the posterior choroid in eyes with central serous chorioretinopathy. *American Journal of Ophthalmology*.

[3] Nicholson B., Noble J., Forooghian F., Meyrele C. (2013). Central serous chorioretinopathy: update on pathophysiology and treatment. *Survey of Ophthalmology*.

[4] Yun C., Oh J., Han J. Y., Hwang S. Y., Moon S. W., Huh K. (2015). Peripapillary choroidal thickness in central serous chorioretinopathy. Is choroid outside the macula also thick?. *Retina*.

[5] Kuroda S., Ikuno Y., Yasuno Y. (2013). Choroidal thickness in central serous chorioretinopathy. *Retina*.

[6] Imamura Y., Fujiwara T., Margolis R., Spaide R. F. (2009). Enhanced depth imaging optical coherence tomography of the choroid in central serous chorioretinopathy. *Retina*.

[7] Liu D. T., Fok A. C., Chan W., Lai T. Y., Lam D. S., Ryan S. J., Schachat A. P., Sadda S. R. (2013). Central serous chorioretinopathy. *Retina*.

[8] Gass J. D. M. (1997). Diseases causing choroidal exudative and hemorrhagic localized (disciform) detachment of the retina and retinal pigment epithelium. *Stereoscopic Atlas of Macular Diseases. Diagnosis and Treatment*.

[9] McClintic S. M., Jia Y., Huang D., Bailey S. T. (2015). Optical coherence tomography angiography of choroidal neovascularization associated with central serous chorioretinopathy. *JAMA Ophthalmology*.

[10] Fung A. T., Yannuzzi L. A., Freund K. B. (2012). Type I (sub-retinal pigment epithelial) neovascularization in central serous chorioretinopathy masquerading as neovascular age-related macular degeneration. *Retina*.

[11] De Carlo T. E., Rosenblatt A., Goldstein M., Baumal C. R., Loewenstein A., Duker J. S. (2016). Vascularization of irregular retinal pigment epithelial detachments in chronic central serous chorioretinopathy evaluated with OCT angiography. *Ophthalmic Surgery, Lasers and Imaging Retina*.

[12] Yannuzzi L. A., Freund K. B., Goldbaum M. (2000). Polypoidal choroidal vasculopathy masquerading as central serous chorioretinopathy. *Ophthalmology*.

[13] El-Maftouhi M. Q., El Maftouhi A., Eandi C. M. (2015). Chronic central serous chorioretinopathy imaged by optical coherence tomographic angiography. *American Journal of Ophthalmology*.

[14] Hage R., Mrejen S., Krivosic V., Quentel G., Tadayoni R., Gaudric A. (2015). Flat irregular retinal pigment epithelium detachments in chronic central serous chorioretinopathy and choroidal neovascularization. *American Journal of Ophthalmology*.

[15] Yang L. H., Jonas J. B., Wei W. B. (2015). Conversion of central serous chorioretinopathy to polypoidal choroidal vasculopathy. *Acta Ophthalmologica*.

[16] Yang L., Jonas J. B., Wenbin W. (2013). Optical coherence tomography-assisted enhanced depth imaging of central serous chorioretinopathy. *Investigative Ophthalmology & Visual Science*.

[17] Stanga P. E., Tsamis E., Papayannis A., Stringa F., Cole T., Jalil A. (2016). Swept-source optical coherence tomography angiography™ (Topcon Corp, Japan): technology review. *Developments in Ophthalmology*.

[18] Bonnin S., Mané V., Couturier A. (2015). New insight into the macular deep vascular plexus imaged by optical coherence tomography angiography. *Retina*.

[19] Grulkowski I., Liu J. J., Potsaid B. (2012). Retinal, anterior segment and full eye imaging using ultrahigh speed swept source OCT with vertical-cavity surface emitting lasers. *Biomedical Optics Express*.

[20] Gao S. S., Liu G., Huang D., Jia Y. (2015). Optimization of the split-spectrum amplitude-decorrelation angiography algorithm on a spectral optical coherence tomography system. *Optics Letters*.

[21] Kuehlewein L., Tepelus T. C., An L., Durbin M. K., Srinivas S., Sadda S. R. (2015). Noninvasive visualization and analysis of the human parafoveal capillary network using swept source OCT optical microangiography. *Investigative Ophthalmology & Visual Science*.

[22] Savastano M. C., Lumbroso B., Rispoli M. (2015). In vivo characterization of retinal vascularization morphology using optical coherence tomography angiography. *Retina*.

[23] Filho M. A. B., De Carlo T. E., Ferrara D. (2015). Association of choroidal neovascularization and central serous chorioretinopathy with optical coherence tomography angiography. *JAMA Ophthalmology*.

[24] Ferrara D., Mohler K. J., Waheed N. (2014). En face enhanced-depth swept-source optical coherence tomography features of chronic central serous chorioretinopathy. *Ophthalmology*.

[25] Maruko I., Iida T., Sugano Y., Ojima A., Sekiryu T. (2011). Subfoveal choroidal thickness in fellow eyes of patients with central serous chorioretinopathy. *Retina*.

[26] Maruko I., Iida T., Sugano Y., Ojima A., Ogasawara M., Spaide R. F. (2010). Subfoveal choroidal thickness after treatment of central serous chorioretinopathy. *Ophthalmology*.

[27] Razavi S., Souied E. H., Cavallero E., Weber M., Querques G. (2014). Assessment of choroidal topographic changes by swept source optical coherence tomography after photodynamic therapy for central serous chorioretinopathy. *American Journal of Ophthalmology*.

[28] Kim Y. T., Kang S. W., Bai K. H. (2011). Choroidal thickness of both eyes of patients with unilaterally active central serous chorioretinopathy. *Eye*.

[29] Teussink M. M., Breukink M. B., Van Grinsven M. J. J. P. (2015). OCT angiography compared to fluorescein and indocyanine green angiography in chronic central serous chorioretinopathy. *Investigative Ophthalmology & Visual Science*.

